# Estimation of the parameters of ETAS models by Simulated Annealing

**DOI:** 10.1038/srep08417

**Published:** 2015-02-12

**Authors:** Anna Maria Lombardi

**Affiliations:** 1 Istituto Nazionale di Geofisica e Vulcanologia, Via di Vigna Murata 605, 00143 Roma, Italy

## Abstract

This paper proposes a new algorithm to estimate the maximum likelihood parameters of an Epidemic Type Aftershock Sequences (ETAS) model. It is based on Simulated Annealing, a versatile method that solves problems of global optimization and ensures convergence to a global optimum. The procedure is tested on both simulated and real catalogs. The main conclusion is that the method performs poorly as the size of the catalog decreases because the effect of the correlation of the ETAS parameters is more significant. These results give new insights into the ETAS model and the efficiency of the maximum-likelihood method within this context.

The Epidemic Type Aftershock Sequences (ETAS) model^17^,^18^ is the most popular stochastic model used to describe earthquake occurrence, to forecast earthquakes and to detect fluid/magma signals or induced seismicity^4^,^9^,^13^,^15^. In spite of its large diffusion, few papers propose procedures to estimate the ETAS parameters or test and discuss the existing methods. Similarly, few codes have been made available to the seismological community on this topic. Moreover, most papers based on ETAS models lack complete information on how to estimate the parameters. Finally, the published algorithms have never been rigorously tested, leading to the conclusion that the ETAS model estimation is anything but straightforward.

A large body of literature exists on the use of ETAS parameters as proxies of processes leading to seismicity^4^,^9^,^13^,^15^. This demands a full investigation of both the significance of the ETAS parameters and the reliability of the maximum likelihood (ML) criterion^18^, which is the most used estimation method.

The first estimation procedure was elaborated by Ogata^17^ for the temporal version of the model. He found the parameters that maximize the log-likelihood by the Davidon-Fletcher-Powell algorithm. The relative Fortran code is included in the software called SASeis2006 and is downloadable from the address http://www.ism.ac.jp/~ogata/Ssg/ssg_softwaresE.html.

This procedure was then extended to the spatio-temporal version of the model by Ogata^18^ and Zhuang et al.^27^,^28^. Two codes were developed from these studies: 1) a Fortran code, written by Zhuang and available at the address http://bemlar.ism.ac.jp/zhuang/software.html, and 2) an R code, written by Jalilian and downloadable from http://cran.r-project.org/web/packages/ETAS/index.html.

After these studies, Veen and Schoenberg^25^ developed an EM (expectation-maximization) algorithm for an accurate estimation of the ETAS model. Their method finds the maximum of the expected complete data log-likelihood, which is based on the probabilistic incorporation of the branching structure. Finally, Lippiello et al.^12^ propose a ML algorithm based on a grid search method. To the best of the author's knowledge the above list is exhaustive.

The original purpose of the present paper is to discuss the efficiency of the ML criterion for estimating the parameters of the ETAS model. As I will show below, this study required repeated runs of an estimation code on a certain amount of simulated catalogs. Algorithms based on numerical methods, such as the one used by Ogata and coworkers, are not suitable for this type of analysis; their performance strongly depends on the fine tuning of some algorithm parameters, which is hard to do automatically. Therefore, I decided to formulate a novel method and to develop a new code. This method is based on Simulated Annealing (SA), which has been found to be useful in a variety of optimization problems, especially those with many independent variables^8^,^21^,^23^. The choice of SA is due to its particular capacity for escaping from local minima/maxima, with respect to other global searching methods. Moreover, it does not need to calculate the partial derivatives, contrary to the gradient and the Newton methods. The main limitation of this technique is that the proper choice of its tuning parameters is mandatory to ensure its effectiveness.

I describe the algorithm in the first two sections of the paper. In the third section, the method is tested on simulated and real catalogs. In the last two sections, I analyze how the SA algorithm performs and discuss some general implications for the ETAS model.

## The Very Fast Simulated Annealing algorithm

SA is a stochastic method to solve problems of multidimensional global optimization, i.e. problems with the following form
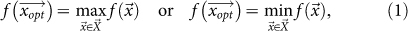
where 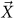 is a *D*-dimensional subset of *R^D^*^8^,^21^,^23^. The term “annealing” refers to a process in which a solid, brought into a liquid phase by increasing its temperature, is brought back to a solid phase by a progressive reduction of the temperature. This process has been done in such a way that all the particles arrange themselves in a perfect crystallized state, representing the global minimum of a certain energy function.

SA algorithms are random iterative procedures that generate a candidate point 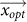 and move to this point or stay at the current one based on a stochastic mechanism. The latter is controlled by the temperature *T*; when decreased, the search becomes more directive.

In the following, I will refer to the case of the global maximization of a function *f*.

More formally, a general SA algorithm can be described as follows.

**Initialization** Generate an initial random solution 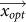. Select a value for the initial temperature *T*_0_ > 0. Set the count *j* = 0.

**Inner loop** Set 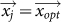 and repeat the following *N_in_* times:

a) generate the next candidate solution 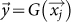;

b) sample a uniformly distributed random number *p* ∈ [0,1] and set 
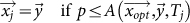


  where *A* is a suitable “acceptance” function;

c) set 
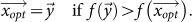


**Outer loop** Check a stopping criterion and,

if satisfied, then STOP;

otherwise

a) set *T_j_*_+1_ = *U*(*T_j_*) ≤ *T_j_* and *j* = *j* + 1;

b) go back to the Inner loop.

In brief, an SA algorithm is an iterative procedure composed of two nested loops. In the outer loop, called the cooling process, the temperature *T* is decreased from its initial value until a convergence criterion is achieved. The inner loop is a random search of a possible better solution 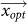 in a region around the local maximum 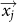.

An SA algorithm applied to a specific problem requires 1) the distribution *G* generating the next candidate point 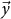; 2) the acceptance function *A* and the number of trials *N_in_* for the inner loop; 3) the initial temperature *T*_0_ and cooling function *U*; and 4) the stopping condition. SA algorithms are conceptually simple, but the setting of these tuning parameters/functions is an extremely tricky and problem-dependent question, crucial for the efficiency of the algorithm (Ref. 19 and references therein). A bad solution to this problem invalidates the effectiveness and robustness of SA procedures, even if they have formal proofs of convergence to the global optima.

### The inner loop: next candidate distribution *G* and acceptance criterion *A*

The function *G* defines the way the model 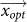 is updated. In brief, it consists of adding a random “perturbation” to the current model 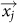 to obtain the new candidate 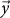. In this study, I adopt the Very Fast Simulate Annealing (VFSA) procedure proposed by Szu and Hartley^22^ and improved by Ingber^5^,^6^. This defines the function *G* as a *D*-dimensional Cauchy distribution such that, for each dimension *k*, the searching region is controlled by the temperature. Specifically
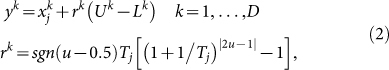
where *L^k^* and *U^k^* are the lower and upper bounds in the *k*th dimension and *u* ∈ [0, 1] is a uniformly distributed random number^5^,^6^,^21^.

The acceptance criterion *A* determines if the new computed solution 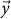 is accepted or discarded. Here, I use the well-known Metropolis criterion^16^,^21^, given by
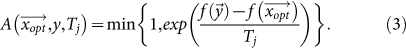
In this way, the ascent steps are all accepted, whereas the descent steps are accepted with a probability controlled by *T_j_*, to not get trapped in local maxima.

### The outer loop: initial temperature *T*_0_, cooling function *U* and the stopping criterion

The initial temperature *T*_0_ and the cooling schedule are of critical importance to the success of SA, especially for a VFSA algorithm, in which the temperature defines both the next candidate 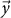 and the acceptance criterion *A* (see eq. 2 and 3). A low *T*_0_ might cause an overly restricted search around the starting point 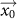. A high *T*_0_ or a slow cooling schedule might cause an overly high computational time and a possible unsuccessful search, especially if the number of iterations *N_in_* is limited. Finally, a fast cooling schedule can trap the algorithm in a local maximum.

Various choices of the initial temperature *T*_0_ have been suggested in the literature (Ref. 1 and references therein). A general rule is that *T*_0_ must be defined in such a way that any solution 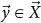 can be selected and almost any model perturbation must be accepted (
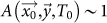
, 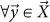) at the beginning. The first condition is satisfied by assuming *T*_0_ ≥ 1, making the distribution of *r_k_* almost uniform (see eq. 2). The second condition depends on the specific problem and can be achieved in different ways (Refs. 1, 7 and references therein). Here, I adopt the simple criterion, suggested by Lin et al.^11^, that expresses *T*_0_ as the ratio between the size of the image of *f* and the number of data.

The cooling schedule regulates how rapidly the temperature *T* varies from high to low values as a function of the iteration count. Here, I apply an Adaptive Cooling method, which adjusts the decrease rate of *T* from information obtained during the algorithm's execution^23^. Specifically, I set
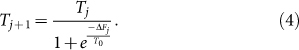
where 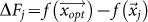.

The idea here is to keep the temperature unchanged when the local maximum 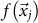 is far from the global optimum 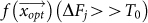 and to half the temperature when the global maximum is updated (Δ*F_j_* = 0).

The algorithm ends when a stopping criterion is satisfied. Two possible rules can be followed: an SA procedure is stopped when *T* decreases up to a pre-selected threshold^24^ or when it does not make significant progress over several iterations^2^. Here, I adopt the second criterion: the algorithm is stopped if its progress over the last *M* iterations is small, i.e., when the following conditions are satisfied for a positive small 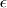:
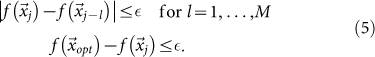


## A VFSA algorithm for estimating the parameters of the ETAS model

In this study, I present a VFSA algorithm to estimate the ML parameters of a magnitude-spatio-temporal ETAS model having intensity
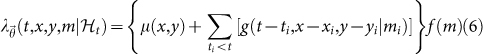
and log-likelihood
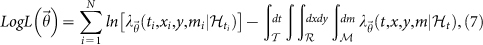
where 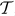, 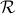 and 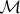 are the temporal interval, the spatial region and the magnitude range covered by the *N* events of the catalog, respectively, and 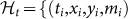 is the history of earthquakes that occurred before *t*.

The algorithm may be adapted to any version of the ETAS model, but I adopt here a specific parameterization for the intensity 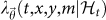.

First, I consider the magnitude distribution 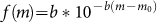
(Gutenberg-Richter law), where *m*_0_ is the completeness magnitude of the catalog. Second, I adopt the triggering function
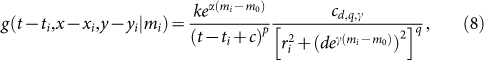
where *c_d_*_,*q*,*γ*_ is the normalization constant of the spatial function and *r_i_* is the distance between the locations (*x*, *y*) and (*x_i_*, *y_i_*). Because the magnitude distribution is independent of the other variables, the b-value can be estimated from magnitudes alone. Therefore, *b* is not included in 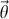.

I model the background rate *μ*(*x*, *y*) by using an equally spaced grid of *N_c_* cells *C_i_*, with central node (*X_i_*, *Y_i_*), covering 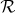. Specifically, I suppose that the background rate is homogeneous inside *C_i_* but variable among the cells such that
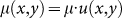
with
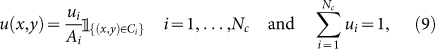
where *u_i_* is the probability of having a background event inside *C_i_* and *A_i_* is the area of *C_i_*. The probabilities *u_i_* are unknown; thus, they belong to the set of parameters 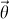. In this first version of the VFSA algorithm, I estimate the probabilities *u_i_* by using the iterative kernel method proposed by Zhuang et al.^27^. In brief, the background distribution is given by
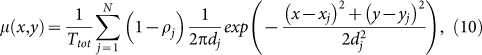
where

- *T_tot_* is the length of the interval time 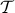;
- *ρ_j_* is the probability that the *j*th event is triggered, given by
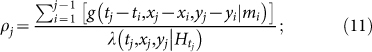
- *d_j_* is a variable bandwidth, given by the radius of the smallest disk, centered at (*x_j_*, *y_j_*), including at least *n_p_* observed events.

Each time the VFSA algorithm updates the best parameters, the probabilities *u_i_* are estimated as
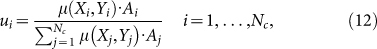
where *μ*(*X_i_*, *Y_i_*) is the background rate, computed at the central node (*X_i_*, *Y_i_*) of the cell *C_i_* by eq. 10. Here, I consider *n_p_* = 10 because the choice of *n_p_* does not significantly affect the results^27^. The log-likelihood calculation requires only the background probabilities of the 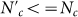 cells with earthquake occurrence (see eq. 2). Thus, the size of 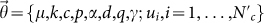 depends on the specific dataset.

Below, I list the VFSA code in detail:

1) Set the count *j* = 0 and the temperature *T* = *T*_0_

- select an initial model 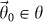 at random and compute 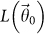
- set 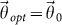 and 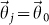

2) Given 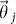

- compute the probability *ρ_j_* for all events using eq. 11
- update the background probabilities *u_i_* for all cells using eq. 12;

3) Repeat the following *N_in_* times

- generate the next candidate set of parameters
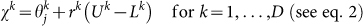
- Compute 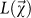 and  if 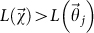, then    
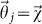
    if 
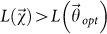
, then 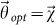
  else  generate a uniformly distributed random number *p* ∈   [0 1]  if 
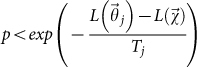
, then 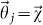
end if

4) if 
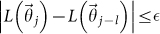
 for 1 = 1,…, *M* and 
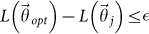
, then   STOP; best parameters 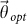
  else

- *j* = *j* + 1
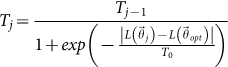
- goto 2

  end if

## Application to ETAS simulated datasets and the Italian real earthquake catalog

**Figure 1:**
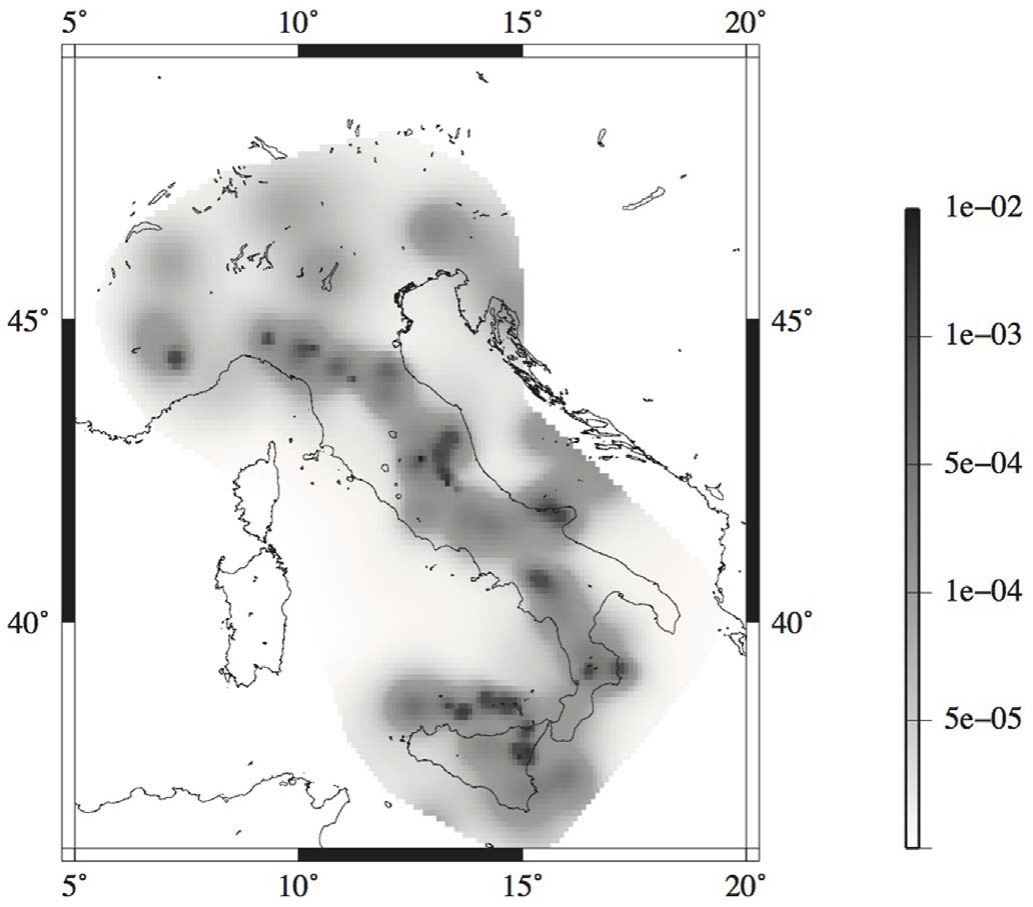
Background spatial probabilities *ui* (see eq. 9) adopted for ETAS simulations. The map was created using the software Generic Mapping Tools (http://gmt.soest.hawaii.edu/).

First, I check the VFSA estimation algorithm on two classes of 100 simulated ETAS datasets, with *T_tot_* = 1 and 5 years, covering the Italian territory. The simulations are obtained by the thinning method^18^, with a *b*-value equal to 1 and the discrete background probability distribution {*u_i_* *i* = 1, …, *N_c_*} shown in Figure 1^14^,^20^. The remaining 8 parameters are randomly selected in the following ranges: *μ* ∈ [0.01 1.0]; *k* ∈ [0.001 0.1]; *p* ∈ [1.0 2.0]; *c* ∈ [0.0001 0.1]; *α* ∈ [0.5 2.0]; *d* ∈ [0.1 1.0]; *q* ∈ [1.0 2.0]; *γ* ∈ [0.0 1.0]. I discard the combination of parameters for which the branching ratio is larger than 1.0 (causing the explosion of the process), and I repeat simulations that give fewer than 100 events. The complementary (or auxiliary) events (i.e., the events occurring outside the spatio-temporal target region 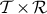 that have a possible triggering effect on the events inside) play a crucial role for correctly estimating the ETAS parameters^26^. Therefore, I take into account the triggering contribution of all the events simulated outside the target region. Moreover, I simulate 1 year of seismicity before the target period 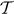 (for both 1 and 5 year catalogs) and I include the triggering contributions of these events in log-likelihood computations. The number of events *N* in the 200 simulated datasets varies from 102 to 3889.

To apply the VFSA algorithm described in the previous section, I fix *N_in_* = 100, and I follow the criterion proposed by Lin et al.^11^ to estimate *T*_0_. For each synthetic catalog, I estimate the log-likelihood's image as the largest difference among the 100 log-likelihood values, computed on as many random combinations of parameters. The values of *T*_0_ obtained in this way vary from 1.0 to 22. Thus, I fix the starting temperature to the mean value *T*_0_ = 10. Finally, I use the conservative value *M* = 10 to define the stopping criterion (see eq. 5), and I fix 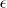, which I judge as an acceptable approximation for the log-likelihood of the ETAS model.

I check the performance of the algorithm by running the VFSA code 100 times on each catalog. Specifically, for each parameter and each catalog, I measure the systematic and the random errors by three quantities: the accuracy, i.e., the closeness of the estimations to the true values, the bias, i.e, the systematic shift of the estimations in one direction from the true values, and the precision, i.e., the degree of agreement for a series of estimations^10^. I measure these quantities as percentages of the size of the range (*RG_k_*) of each parameter 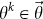, because the ETAS parameters have different orders of magnitude. The bias of *θ^k^* is measured by:
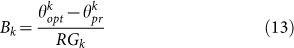
where 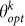 is the median of the output estimations of 100 runs 
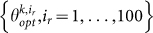
 and 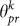 is the pseudo-real value of *θ^k^* (i.e. the value used to simulate the dataset). I compute the accuracy as the absolute value of the bias:
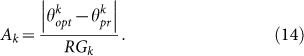
Finally, the precision is given by:
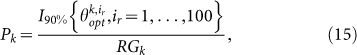
where 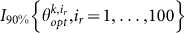 is the size of the 90% confidence interval for the estimated *θ^k^*, computed as the difference between the 95-th and 5-th percentiles of 
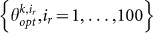
.

**Figure 2:**
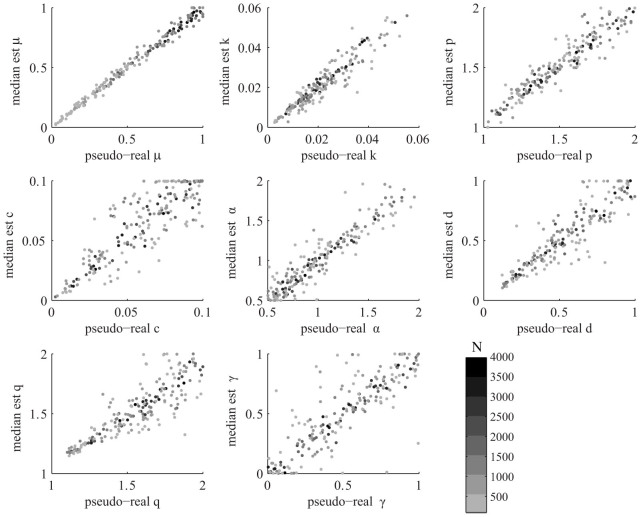
Plot of median estimates 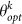 versus the pseudo-real values 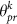 for the 8 parameters of the ETAS model {*μ*, *k*, *p*, *c*, *α*, *d*, *q*, *γ*} and for all the simulated catalogs. The color is scaled with the size of the catalog (see text for details).

**Figure 3:**
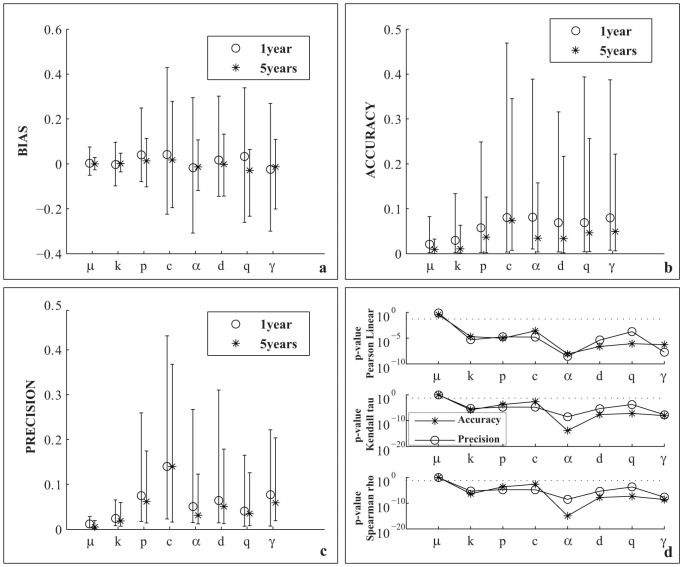
Analysis of bias, accuracy and precision of ML estimation of the ETAS parameters, obtained by the VFSA algorithm proposed in the present study. (a) Distribution of the bias for all the simulated ETAS catalogs and for the 8 parameters of the ETAS model. Symbols mark the median values (circles for 1 year catalogs, stars for 5 year catalogs). The bounds show the 5-th and the 95-th percentiles (of the values obtained for each catalog). (b) The same of (a), except for accuracy. (c) The same of (a), except for precision. (d) Results of statistical tests applied to check the correlation/dependence between the number of earthquakes *N* and the accuracy/precision of the 8 parameters of the ETAS model. The hypothesis of independence is rejected for all parameters, except for *μ*.

**Figure 4:**
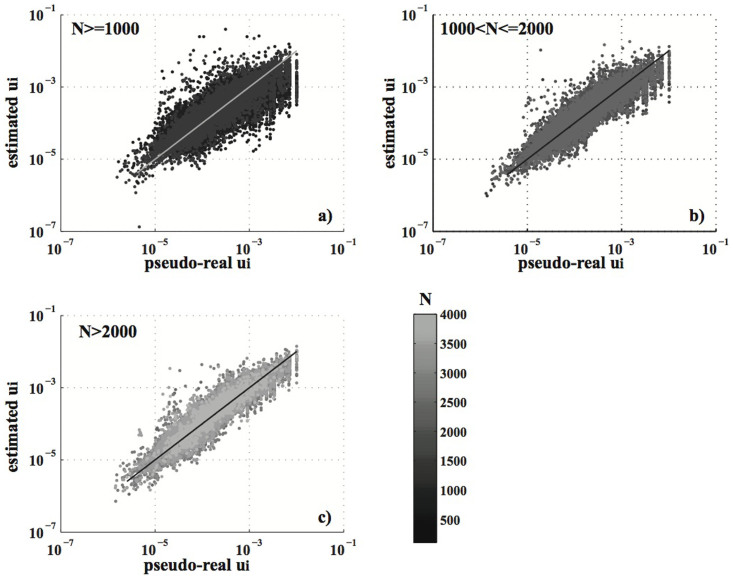
Plot of the estimated median (on 100 runs) background probabilities *u_i_* versus the pseudo real values for all cells with at least an event and for all catalogs. The color is scaled with the size of the catalog *N*. (a) Plot for catalogs with *N* ≤ 1000. (b) The same as a), but for 1000 < *N* ≤ 2000. (c) The same as (a), but for *N* > 2000.

Figure 2 shows the plot of 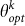 versus 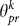 for the 8 parameters {*μ*, *k*, *c*, *p*, *α*, *d*, *q*, *γ*} and for all simulated datasets. 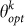 and 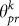 may significantly differ, especially for small datasets, except for the parameter *μ*. Figure 3 is a synthesis of the bias/accuracy/precision measures. It shows no systematic significant bias (under or over-estimation); the distribution of *B_k_* is centered at zero and is mainly symmetric (see Figure 3a) for all parameters. *A_k_* is well below 0.1 for more than 50% of the catalogs, but it reaches 0.4–0.5 for the *c* parameter (see Figure 3b). Similarly, the precision is below 20–30% of *RG_k_* many times but may reach 40% for the 1-year catalogs (see Figure 3c). A possible proxy of the accuracy/precision of the algorithm is the number of events *N*. Thus, I test the hypothesis of no correlation/dependence between *A_k_*/*P_k_* and *N* by three statistic tests: the Pearson's linear, the Spearman rho and the Kendall's tau coefficients^3^. The first is a measure of the linear relationship between two samples, while the others quantify a more general association. The results are shown in Figure 3d. The p-values of the tests (i.e. the probabilities of non-correlation/independence) are well below 0.01 for all parameters, except for *μ*, suggesting a strong influence of *N* on the estimation. The high p-values for the *μ* parameter confirm the accuracy/precision results (see Figures 2 and 3): the algorithm is able to estimate the overall background rate, no matter how large the dataset is. In contrast, the sample size *N* strongly affects the fit of the spatial probability background distribution (Figure 4). The medians (on 100 runs, for each catalog) of the estimated probabilities *u_i_* are closer to the pseudo-real values (mapped in Figure 1) as *N* increases. For small datasets, the estimated and pseudo-real probabilities may differ by as much as two orders of magnitude.

**Figure 5:**
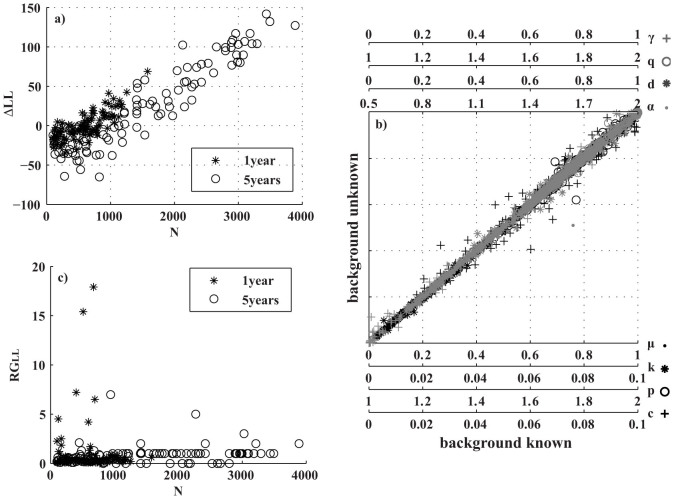
Analysis of log-likelihood values. (a) Difference of log-likelihoods, computed on the pseudo-real and the estimated parameters (Δ*LL*), as a function of the size of the catalog (*N*). (b) Plot of the estimated values of the 8 parameters of the ETAS model, obtained by assuming the background probabilities *u_i_* known/unknown. The x and y axes are equal and are scaled with the range of each parameter. On the right of each x-axis, the corresponding parameter and symbol are reported. (c) Range of variability of log-likelihoods (*RG_LL_*, eq.16) versus the number of events (*N*) for all catalogs.

The analysis of log-likelihood values confirms the previous results (see Figure 5). First, the values 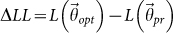 are negative for *N* below 1000/1500 (see Figure 5a), suggesting that the data are not sufficient to optimize the log-likelihood. If the background probabilities *u_i_* are fixed to pseudo-real values, Δ*LL* is always positive, varying from 0 to 20, with negligible differences for the remaining 8 parameters (see Figure 5b). This means that the negative values of Δ*LL* are entirely due to the poor estimation of the background probabilities *u_i_*. The largest difference among the log-likelihoods, deduced from the best 100 runs as follows:
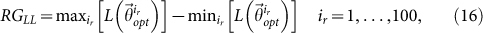
is mainly below 2 (see Figures 5c). This suggests that the low precision and accuracy of the estimations on the smallest catalogs are due to the flatness of the log-likelihood function. In other words, rather different combinations of parameters (see Figure 3c) may give similar log-likelihood values on small datasets.

I further investigate this point by applying the three tests used in Section 3 on all possible^28^ pairs of 100 parameter values obtained by as many runs. Specifically, I compute the proportion of catalogs with p-values < 0.05 for each pair of parameters. The aim of this analysis is to verify the hypothesis of non-correlation/independence among the ETAS parameters. I find significant correlations inside two parameter subsets: {*k*, *c*, *p*, *α*} (Omori law) and {*d*, *q*, *γ*} (spatial distribution of triggered events). No systematic correlation is found between *μ* and the other parameters, confirming that the algorithm is able to distinguish the background seismicity.

To clarify the procedure, I describe in more details the results of the algorithm for the smallest and the largest simulated catalogs (see Tables 1 and 2). The first catalog has 1 year of data (plus 1 year as an auxiliary period) and 102 events; the second has 3889 events collected over a five-year period (plus 1 year as an auxiliary period). The solution for the first dataset has poor precision (such as for the *c* parameter) and poor accuracy (the *α* parameter), but the values 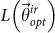 are close and all lower than the pseudo-real value 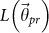. No significant change is found by varying some tuning parameters of the algorithm (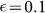; *T*_0_ = 1, 100; *n_p_* = 5). If I fix the background probabilities to pseudo-real values, the algorithm gives similar estimations of the remaining 8 parameters and log-likelihood values larger than 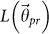 and close to −662.0. This confirms that the log-likelihood values strongly depend on the background probabilities. In contrast, the parameter estimation on the second catalog is accurate and precise; this proves that the algorithm provides correct solutions on sufficiently large datasets.

I repeated the analysis on simulations with an extended auxiliary period (up to 5 years), but did not find significant changes.

Finally, I apply the algorithm presented here on real data as a further check. Specifically, I consider the events that occurred in the last 10 years or so (16/04/2005-31/08/2014) in the region shown in Figure 1 with local magnitude larger than 3.0 and depth lower than 30 km. This database is down-loadable from the site http://iside.rm.ingv.it and consists of 1788 events. It includes two important sequences, which occurred near L'Aquila city (2009, stronger event ML5.9) and in the Emilia region (2012, stronger event ML5.9). I run the VFSA algorithm 100 times, obtaining the median values and the 90% confidence bounds of the ETAS parameters, which are reported in Table 3. The comparison with the output estimations of the code developed by Zhuang and co-workers is not trivial, because they adopt a different formulation of the model with a normalized Omori law^27^,^28^. However, I find negligible differences by adapting the best parameters found with Zhuang's code to my formulation (see Table 3).

## Discussions and Conclusions

In this study, I present a new algorithm to estimate the parameters of a spatio-temporal ETAS model. The algorithm is based on SA and allows an automatic ML estimation of the model, without ad-hoc tuning of the parameters. Moreover, it quantifies the uncertainties by multiple runs. It is a versatile and testable tool, used here to investigate the model and the possibility to give a physical interpretation to the parameters. This type of study needs repeated simulations and estimations, which are difficult to do using conventional ML procedures. Numerical optimization procedures may be computationally intensive or may have some convergence problems, especially when the log-likelihood is extremely flat or multimodal. Moreover, it is quite difficult to decide if log-likelihoods have converged to the global maximum. Finally, their performance strongly depends on the starting values of the parameters.

I test the algorithm proposed here on two classes of 100 synthetic datasets: 1 and 5 years (plus 1 year of an auxiliary period, see Ref. 26); these are simulated by using a specific formulation of ETAS model and randomly generated sets of parameters (see Section 3). The choice of testing the algorithm on relatively small datasets is intentional. The efficiency of the ML criterion on large catalogs is expected, if a proper auxiliary region is taken into account^26^. However, it is still unexplored if the ML criterion is able to discern the ETAS parameters on small catalogs, even if such datasets are largely used in studies on non-stationarities^4^,^9^,^13^.

**Figure 6:**
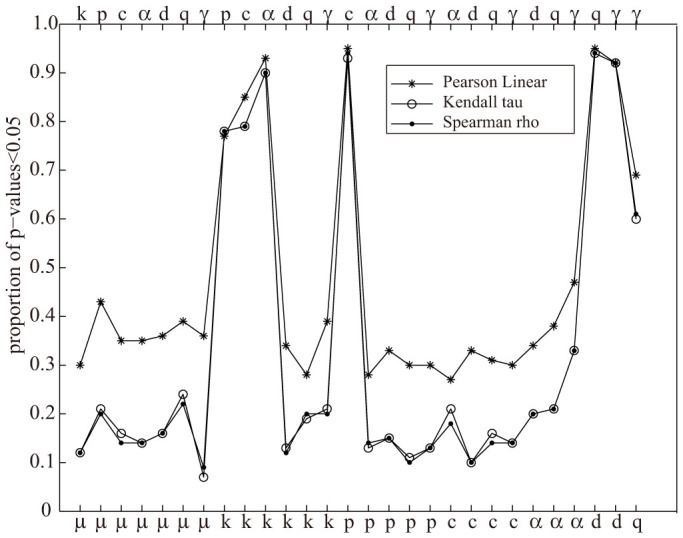
Analysis of dependence/correlation for all possible pairs of the 8 parameters of the ETAS model. The probability of independence/non-correlation is computed on values obtained by 100 runs for each catalog, each pair of parameters and each statistical test. The proportion of catalogs with p-values < 0.05 is plotted as a function of the pairs of parameters (the parameters are labeled on the top and bottom x-axes).

The results of this study consistently show that a few data (below 1000) do not produce a precise and correct estimation of the model; only the overall background rate is correctly distinguished from the triggering contribution, regardless of the number of data. In particular, the algorithm is not able to give a reliable estimation of the discrete background probabilities *u_i_* for small datasets. This is the only cause for the failure in the log-likelihood optimization (see Figure 5a) and does not affect the other parameters (see Figure 5b). These inefficiencies may derive from the specific procedure used here, the inappropriateness of the ML criterion itself, as outlined by Veen and Schoenberg^25^, or the intrinsic impossibility of estimating so many parameters with a few events. This will be the topic of a future communication. What is certain is that rather different combinations of parameters may give close log-likelihoods on small datasets (see Figure 5c and Tables 1–2); this is the main cause of the low precision of the algorithm and it is in part due to the high correlation among the parameters of the triggering contribution (Figure 6).

**Figure 7:**
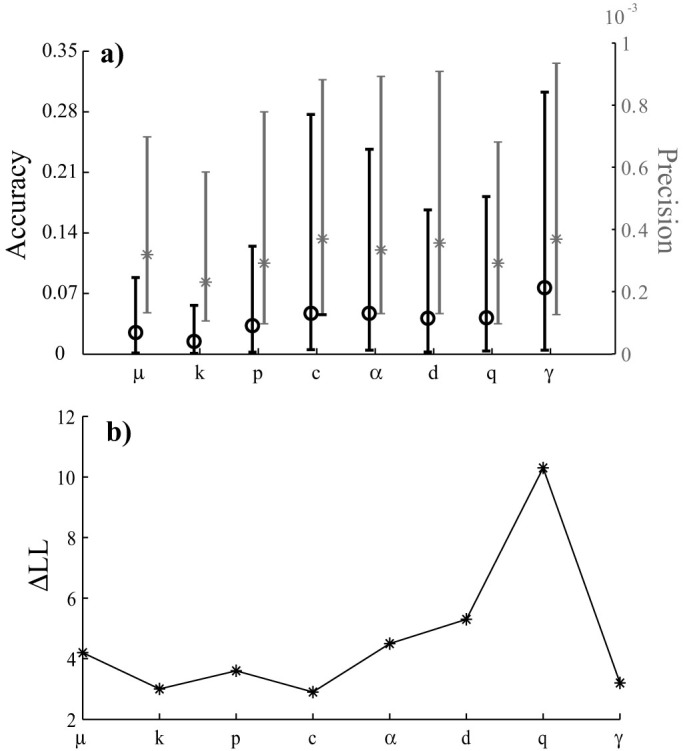
“Partial” estimation of the ETAS model on 1-year simulated catalogs. Each parameter is estimated one at a time by keeping the remaining parameters and the background probabilities fixed to the pseudo-real values. (a) Distribution of accuracy and precision. The symbols mark the median values (circles for accuracy, stars for precision). The bounds indicate the 5-th and the 95-th percentiles (of the values obtained for each catalog). (b) Difference of the median log-likelihoods computed on pseudo-real and estimated parameters (Δ*LL*), as a function of the “free” parameter. Due to the high precision of this type of estimation, the log-likelihoods of 100 runs for each catalog are close.

This study gives important hints on the ETAS model as a diagnostic tool of the physical processes responsible for seismicity. First, it shows that the spatial-temporal variability of the *μ* parameter may be a proxy of the magma/fluid signal^4^,^13^ or of induced seismic activity^9^ because the ML method allows the background rate to be distinguished from the triggering contribution. However, any speculation on spatio-temporal variations of the triggering contribution must be treated with the utmost care. This topic needs more work, of course. However, to give more arguments, I conduct a “partial” estimation on the 1-year catalogs. Specifically, I estimate each parameter one at a time, keeping both the remaining parameters and the pseudo-real values of the background probabilities fixed (Figure 7). The resulting accuracy does not differ much from the one obtained from a “full” analysis (see Figure 3), for which the precision is much higher (Figure 7a) and the log-likelihood values are always larger than 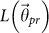 (Figure 7b). All these results suggest two points: first, the low precision of the “full” estimation (Figure 3c) is mainly due to the correlation of parameters; second, the low accuracy (Figures 3b and 7a) derives from the low resolution of the ETAS model and/or of the ML criterion on small datasets. In particular, the limited magnitude ranges, covered with small datasets, make the estimations of the *α* and *γ* parameters inaccurate.

Different ETAS estimation methods might clarify if all these problems are intrinsic to the model or due to the flatness of log-likelihood. Until now, the only possible alternative is the EM-method of Veen and Schoenberg^25^, proposed as a better method than the ML criterion, especially on limited datasets. This does not mean that the ML criterion is wrong, but that the quality and amount of experimental data may be too poor to estimate the parameters unambiguously. And, if the model parameters are not well set, then all the points inferred from their estimation are misleading.

The procedure proposed here allows an estimation of ETAS parameters without an ad hoc tuning of the algorithm, as the Quasi-Newton methods require. Moreover, multiple runs quantify the errors. The results on the real Italian dataset show that the uncertainties are not symmetric (see Table 3). Therefore, the Hessian matrix^18^ may wrongly estimate the errors, because it needs a normal distribution of the uncertainties.

In the present study, strong attention is not paid to cutting the computational time of the algorithm. However, I judge it reasonable in this first version, also for large datasets. Parallelization and/or some changes would make the code faster. This will be the topic of future work.

## Figures and Tables

**Table 1:** Results of model estimations for the smallest simulated catalog. The first column lists the pseudo-real parameters. In the second column, the median (on 100 runs) estimated values are reported. The third and the fourth columns list the log-likelihood values for pseudo-real and estimated parameters. The fifth column reports the expected number of events. The values in brackets are the 5-th and the 95-th percentiles of relative variables. The numbers in boldface mark the results obtained by fixing the background probabilities *u_i_* to pseudo-real values. The last column lists significant iterations of the run giving the higher maximum likelihood (the first 8 values, in curly braces, are the parameters; the last is the log-likelihood)

1 year, 102 events					
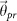	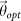	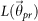	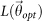	Nev	Run Hist
*μ* = 9.74E−2	*μ* = 1.06 (1.03, 1.08)E−1	−665.3	−682.6 (−682.7, −682.6)	102 (101, 103)	{5.43E−2, 1.19E−2, 1.64, 8.95E−2, 0.98, 0.44, 1.34, 0.44}; −694.0
	***μ* = 1.06 (1.04, 1.08)E−1**		**−661.9 (−662.2, −661.9)**	102 (101, 103)	{7.41E−2 4.76E−3 1.66 7.19E−2 1.07 0.75 1.79 0.82}; −687.7
*k* = 1.68E−2	*k* = 4.96 (4.19, 6.00)E−3				{0.14, 4.70E−3, 1.16, 8.64E−2, 1.53, 0.71, 1.87, 0.75}; −687.6
	***k* = 4.90 (4.06, 6.72)E−3**				{0.13, 4.48E−3, 1.55, 8.77E−2, 1.82, 0.85, 1.32, 0.43}; −685.7
*p* = 1.21	*p* = 1.48 (1.40, 1.53)				{9.40E−2, 5.94E−3, 1.44, 4.25E−2, 1.59, 0.90, 1.67, 0.80}; −683.7
	***p* = 1.51 (1.39, 1.56)**				{9.37E−2, 5.110E−3, 1.52, 6.75E−2, 1.70, 0.87, 1.88, 0.78}; −683.4
*c* = 5.05E−2	*c* = 7.95 (5.48, 9.50)E−2				{0.10, 5.88E−3, 1.46, 6.26E−2, 1.82, 0.83, 1.85, 0.77}; −683.2
	***c* = 8.23 (4.86, 9.83)E−2**				{0.10, 4.55E−3, 1.46, 6.17E−2, 1.82, 0.84, 1.76, 0.77}; −683.1
*α* = 7.94E−1	*α* = 1.90 (1.77, 1.97)				{0.10, 4.60E−3, 1.48, 7.46E−2, 1.80, 0.83, 1.82, 0.90}; −683.1
	***α* = 1.88 (1.70, 1.96)**				{0.10, 6.21E−3, 1.47, 7.44E−2, 1.90, 0.93, 1.84, 0.83}; −683.1
*d* = 8.78E−1	*d* = 9.96 (9.69, 9.99)E−1				{0.10 6.02E−3 1.49 7.42E−2 1.89 0.97 1.83 0.79}; −683.0
	***d* = 9.97 (9.61, 9.99)E−1**				{0.10, 5.06E−3, 1.48, 7.36E−2, 1.89, 0.97, 1.84, 0.79}; −682.9
*q* = 1.62	*q* = 1.98 (1.93, 1.99)				{0.11, 5.19E−3, 1.47, 7.80E−2, 1.91, 0.96, 1.84, 0.81}; −682.9
	***q* = 1.98 (1.85, 1.99)**				{0.10, 4.48E−3, 1.47, 6.26E−2, 1.91, 0.96, 1.84, 0.81}; −682.8
*γ* = 5.52E−1	*γ* = 7.46 (6.99, 7.94)E−1				{0.10, 4.43E−3, 1.46, 6.62E−2, 1.91, 0.96, 1.95, 0.81}; −682.7
	***γ* = 7.44 (7.03, 7.82)E−1**				{0.11, 4.70E−3, 1.46, 6.63E−2, 1.90, 0.94, 1.97, 0.81}; −682.7
					{0.11, 4.70E−3, 1.49, 7.77E−2, 1.90, 0.94, 1.97, 0.81}; −682.7
					{0.11, 4.80E−3, 1.47, 7.80E−2, 1.92, 0.99, 1.97, 0.81}; −682.6
					{0.11, 4.94E−3, 1.48, 7.97E−2, 1.86, 0.98, 1.98, 0.76}; −682.6
					{0.10, 4.91E−3, 1.49, 8.01E−2, 1.87, 0.98, 1.98, 0.76}; −682.6
					{0.11, 4.54E−3, 1.49, 8.07E−2, 1.92, 0.99, 1.99, 0.74}; −682.6

**Table 2:** Results of model estimations for the largest simulated catalog. The first column lists the pseudo-real parameters. In the second column, the median (on 100 runs) estimated values are reported. The third and the fourth columns list the log-likelihood values for pseudo-real and estimated parameters. The fifth column reports the expected number of events. The values in brackets are the 5-th and the 95-th percentiles of relative variables. The last column lists significant iterations of the run giving the higher maximum likelihood (the first 8 values, in curly braces, are the parameters; the last is the log-likelihood)

5 years, 3889 events					
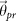	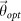	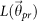	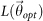	Nev	Run Hist
*μ* = 9.40E–1	*μ* = 9.67 (9.38, 9.74)E–1	−31145	−31018 (−31020, −31017)	3890 (3865,3905)	{0.77, 1.68E−2, 1.85, 4.35E−2, 0.89, 0.90, 1.26, 2.98E−2}; −31725.0
*k* = 2.57E–2	*k* = 2.60 (2.49, 2.76)E–2				{0.56, 3.68E−2, 1.54, 6.25E−2, 0.84, 0.58, 1.86, 0.69}; −31343.1
*p* = 1.71	*p* = 1.73 (1.69, 1.78)				{0.91, 3.50E−2, 1.38, 7.60E−2, 1.08, 0.58, 1.83, 0.29}; −31325.7
*c* = 7.38E–2	*c* = 7.81 (7.04, 8.86)E–2				{0.95, 6.23E−2, 1.56, 9.76E−2, 0.58, 0.90, 1.96, 0.30}; −31228.5
*α* = 1.26	*α* = 1.24 (1.21, 1.26)				{0.98, 2.95E−2, 1.85, 9.39E−2, 1.36, 0.68, 1.47, 0.50}; −31227.4
*d* = 7.95E–1	*d* = 7.18 (6.77, 7.84)E–1				{0.96, 5.17E−2, 1.48, 8.41E−2, 0.97, 0.73, 1.56, 0.24}; −31197.4
*q* = 1.88	*q* = 1.77 (1.74, 1.83)				{0.95, 3.42E−2, 1.47, 4.39E−2, 1.20, 0.78, 1.53, 0.27}; −31174.1
*γ* = 3.98E–1	*γ* = 3.97 (3.69, 4.24)E–1				{0.83, 2.24E−2, 1.45, 2.93E−2, 1.27, 0.78, 1.62, 0.18}; −31110.2
					{0.91, 2.37E−2, 1.55, 5.38E−2, 1.20, 0.75, 1.62, 2.40E−2}; −31094.5
					{0.91, 2.21E−2, 1.53, 4.57E−2, 1.20, 0.79, 1.62, 4.25E−2 }; −31088.3
					{0.87, 2.56E−2, 1.53, 5.29E−2, 1.24, 0.75, 1.83, 0.57}; −31067.4
					{0.86, 2.52E−2, 1.53, 5.38E−2, 1.14, 0.72, 1.83, 0.51}; −31062.8
					{0.94, 2.51E−2, 1.50, 5.35E−2, 1.32, 0.64, 1.78, 0.52}; −31055.3
					{0.93, 2.24E−2, 1.61, 5.18E−2, 1.35, 0.64, 1.80, 0.52}; −31041.4
					{0.94, 2.11E−2, 1.65, 6.13E−2, 1.32, 0.64, 1.74, 0.52}; −31032.5
					{0.93, 2.27E−2, 1.72, 7.10E−2, 1.28, 0.61, 1.72, 0.48}; −31026.4
					{0.99, 2.32E−2, 1.73, 7.27E−2, 1.29, 0.60, 1.72, 0.48}; −31025.4
					{0.99, 2.53E−2, 1.74, 7.97E−2, 1.28, 0.67, 1.78, 0.45}; −31020.3
					{0.97, 2.52E−2, 1.74, 8.03E−2, 1.27, 0.69, 1.76, 0.42}; −31018.2
					{0.97, 2.58E−2, 1.74, 7.95E−2, 1.25, 0.71, 1.76, 0.39}; −31017.0

**Table 3:** Estimation of ETAS parameters for the Italian catalog. The results obtained by using the VFSA and the Quasi-Newton algorithms are compared. For the VFSA algorithm, the median (on 100 runs) values are reported. The values in the brackets are the 5-th and the 95-th percentiles of the relative parameter

Parameter	VFSA algorithm	Quasi-Newton algorithm
*μ*	2.0 (2.0, 2.1) ·10^−1^	2.1 ·10^−1^
*k*	2.3 (2.2, 2.6) ·10^−2^	2.2 ·10^−2^
*p*	1.13 (1.12, 1.15)	1.15
*c*	7.0 (6.0, 9.0) ·10^−3^	8.0 ·10^−3^
*α*	1.4 (1.4, 1.5)	1.5
*d*	9.3 (9.0, 9.7) ·10^−1^	8.8 ·10^−1^
*q*	1.78 (1.75, 1.80)	1.78
*γ*	4.8 (4.4, 4.9) ·10^−1^	4.9 ·10^−1^
Nev	1789 (1777, 1806)	1793
